# Long non‐coding RNA growth arrest‐specific 5 and its targets, microRNA‐21 and microRNA‐140, are potential biomarkers of allergic rhinitis

**DOI:** 10.1002/jcla.23938

**Published:** 2021-09-02

**Authors:** Ji Song, Taojiao Wang, Yandan Chen, Ruixiang Cen

**Affiliations:** ^1^ Department of Otorhinolaryngology The University‐Town Hospital of Chongqing Medical University Chongqing China; ^2^ Department of Otorhinolaryngology Huangshi Central Hospital Affiliated Hospital of Hubei Polytechnic University Edong Healthcare Group HuangShi China

**Keywords:** allergic rhinitis, long non‐coding RNA growth arrest‐specific 5, microRNA‐140, microRNA‐21, Th1&Th2 cells

## Abstract

**Objective:**

Long non‐coding RNA growth arrest‐specific 5 (lnc‐GAS5) and its targets (microRNA [miR]‐21 and miR‐140) are involved in the development and progression of allergic rhinitis (AR). However, the correlation of lnc‐GAS5 with miR‐21 and miR‐140 and their associations with disease risk, symptom severity, and Th1/Th2 cytokines in AR remain unclear. Thus, this study aimed to investigate this topic.

**Methods:**

In total, 120 patients with AR and 60 controls were recruited. Nasal‐mucosa tissues were collected from all participants. Lnc‐GAS5, its targets (miR‐21 and miR‐140), interferon (IFN)‐γ, interleukin (IL)‐2, IL‐4, and IL‐10 were detected by reverse‐transcription quantitative polymerase chain reaction.

**Results:**

Lnc‐GAS5 was elevated, while miR‐21 and miR‐140 was downregulated in AR patients than in controls (*p* < 0.001). In AR patients, lnc‐GAS5 was negatively correlated with miR‐21 (*p* < 0.001), miR‐140 (*p* < 0.001), IFN‐γ (*p* = 0.019), and IL‐2 (*p* = 0.039) and positively correlated with IL‐4 (*p* = 0.004) and IL‐10 (*p* < 0.001), individual nasal symptom scores (INSSs) for itching, sneezing, and congestion (*p* < 0.05), and total nasal symptom score (TNSS) (*p* < 0.001). Moreover, miR‐21 and miR‐140 were negatively correlated with some INSSs, total TNSS score, and IL‐10 and positively correlated with IFN‐γ and IL‐2 (*p* < 0.05).

**Conclusion:**

Lnc‐GAS5 is negatively correlated with that of its targets (miR‐21 and miR‐140) in AR; meanwhile, lnc‐GAS5, miR‐21, and miR‐140 are correlated with disease risk, symptom severity, and Th1/Th2 imbalance in AR, suggesting the potential of these biomarkers in the development and progression of AR.

## Graphical abstract

Objective: Long non‐coding RNA growth arrest‐specific 5 (lnc‐GAS5) and its targets (microRNA (miR)‐21 and miR‐140) take part in the pathology of allergic rhinitis (AR) development and progression. However, the correlation of lnc‐GAS5 with miR‐21 and miR‐140, as well as their associations with disease risk, severity, and Th1/Th2 cytokines of AR is unclear. Thus, the aim of this study was to investigate this topic.Methods: Totally, 120 AR patients and 60 controls were recruited. Besides, nasal mucosa tissues were collected from all participants. Then, lnc‐GAS5, its targets (miR‐21 and miR‐140), interferon (IFN)‐γ, interleukin (IL)‐2, IL‐4, and IL‐10 were detected by reverse‐transcription quantitative polymerase chain reaction.Results: Lnc‐GAS5 was elevated, while miR‐21 and miR‐140 were downregulated in AR patients compared to controls (all *p *< 0.001). In AR patients, lnc‐GAS5 was negatively correlated with miR‐21 (*p *< 0.001), miR‐140 (*p *< 0.001), IFN‐γ (*p *= 0.019), and IL‐2 (*p *= 0.039), while positively correlated with IL‐4 (*p *= 0.004) and IL‐10 (*p *< 0.001), as well as some of individual nasal symptom score (INSS) scores (itching, sneezing, and congestion scores) (all *p *< 0.05) and total nasal symptom score (TNSS) score (*p *< 0.001). Moreover, both miR‐21 and miR‐140 were negatively correlated with some of INSS scores, TNSS score, and IL‐10, while positively correlated with IFN‐γ and IL‐2 (all *p *< 0.05).Conclusion: Lnc‐GAS5 is negatively correlated with its targets (miR‐21 and miR‐140) in AR; meanwhile, they are all correlated with disease risk, symptom severity, and Th1/Th2 imbalance of AR, suggesting the potential of lnc‐GAS5, miR‐21, and miR‐140 as biomarkers for development and progression of AR.

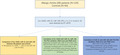

## 1 INTRODUCTION

Allergic rhinitis (AR), an immune disease characterized by immunoglobulin E (IgE)‐related inflammation in the nasopharynx, affects nearly a quarter of the global population.^1^, ^2^, ^3^ The imbalance in innate and adaptive immunity as well as genetic elements play crucial roles in the pathogenesis of AR.^4^ Currently, the treatment of AR generally includes targeted pharmacotherapy and allergen immunotherapy, which can provide a certain measure of symptomatic relief for AR‐related rhinorrhea, itching, and sneezing; however, AR remains challenging to cure.^3^, ^4^, ^5^ AR is usually accompanied by various comorbidities, such as headache, asthma, and atopic dermatitis, which particularly affect the quality of life of AR patients.^1^, ^5^ Because the morbidity of AR has continued to increase the past decades and has led to an increased economic burden worldwide, the exploration of disease‐related biomarkers might be crucial for improving the management of AR.^1^, ^4^

Long non‐coding RNAs (lncRNAs) participate in various biological processes, such as the immune response and inflammatory regulation.^6^ Among well‐known functional lncRNAs, lncRNA growth arrest‐specific 5 (lnc‐GAS5) has been reported to play an important role in regulating the differentiation of T cells in diseases associated with immune dysfunction.^7^, ^8^, ^9^ For instance, lnc‐GAS5 can regulate the differentiation of helper T cell (Th) 1 and Th2, which are involved in the pathogenesis of AR.^7^ Furthermore, several studies have illustrated that lnc‐GAS5 can modulate the differentiation of CD4^+^ T cells in patients with HIV and the imbalance in Th1 and Th2 in patients with recurrent pregnancy loss by targeting microRNA (miR)‐21 and miR‐140‐5p, respectively.^10^, ^11^ Lnc‐GAS5 has been demonstrated to bind directly to miR‐21 and miR‐140‐5p at the molecular level^10^, ^11^; moreover, both miR‐21 and miR‐140‐5p have been correlated with the development of AR.^12^, ^13^ Therefore, we speculated that lnc‐GAS5 and its targets (miR‐21 and miR‐140) might be potential biomarkers of AR. However, information on this association is limited.

Therefore, herein, we explored the correlation of lnc‐GAS5 with miR‐21 and miR‐140 and their association with disease risk, symptom severity, and Th1/Th2 cytokines in AR.

## 2 MATERIALS AND METHODS

### 2.1 Patients and controls

This study was approved by the Institutional Review Board. In total, 120 patients with AR were consecutively enrolled between March 2019 and June 2020. The main inclusion criteria were as follows: (1) diagnosis of AR based on the criteria of the Chinese Society of Allergy Guidelines for Diagnosis and Treatment of Allergic Rhinitis^14^; (2) age >18 years; and (3) available nasal‐mucosa tissue samples. The exclusion criteria were as follows: (1) presence of other concomitant respiratory illnesses such as bronchial asthma, nasal polyposis, or chronic obstructive pulmonary disease (COPD); (2) active infections; (3) history of solid tumor, inflammatory disease, or autoimmune diseases, including rheumatoid arthritis and systemic lupus erythematosus; and (4) lactating or pregnant women. In addition, 60 snoring patients without allergic symptoms were recruited as controls on meeting the following criteria: (1) no history of AR, asthma, or COPD; (2) no concomitant inflammatory diseases, autoimmune diseases, infections, or solid tumors; and (3) available nasal‐mucosa tissue samples. All participants provided written informed consent.

### 2.2 Data and specimen collection

After recruitment, the characteristics of all participants were recorded. After the participants provided informed consent, nasal‐mucosa tissue samples were collected under local anesthesia, washed using normal saline, and sliced into pieces. RNAlater^®^ (Sigma‐Aldrich, Burlington, MA, USA) was added to the specimens to protect the RNA samples from degradation by RNase. Finally, the specimens were stored at −70°C for subsequent detection.

### 2.3 Disease severity assessment

The individual nasal symptom score (INSS) was used to assess disease severity in AR patients. The INSS comprises scores for four symptoms, namely rhinorrhea, sneezing, itching, and congestion. The detailed scoring method was as follows: 0, no symptoms; 1, mild symptoms; 2, moderate symptoms; and 3, severe symptoms. The total nasal symptom score (TNSS) was calculated as the sum of the scores of the four symptoms and ranged from 0 to 12. A higher TNSS indicated a more severe disease.

### 2.4 Reverse‐transcription quantitative polymerase chain reaction (RT‐qPCR)

The expression of lnc‐GAS5, miR‐21, and miR‐140 and the expression of Th1 cell‐secreted cytokines (interferon [IFN]‐γ and interleukin [IL]‐2) and Th2 cell‐secreted cytokines (IL‐4 and IL‐10) were determined by RT‐qPCR. Briefly, total RNA was extracted using TRIzol™ Reagent (Thermo Fisher Scientific, Waltham, MA, USA) and reverse transcribed using the iScript™ cDNA Synthesis Kit (Bio‐Rad, Hercules, CA, USA). Afterward, qPCR was performed using THUNDERBIRD^®^ SYBR^®^ qPCR Mix (Toyobo, Osaka, Kansai, Japan). The primers used in RT‐qPCR are listed in Table S1; GAPDH was used as an internal reference for lncRNAs and mRNAs and U6 for miRNAs. Moreover, the relative expression of lnc‐GAS5, miR‐21, miR‐140, IFN‐γ, IL‐2, IL‐4, and IL‐10 was calculated using the 2^−ΔΔCt^ method.

### 2.5 Statistical analysis

Continuous variables and categorical variables were presented as mean with standard deviation (SD), median with interquartile range (IQR), or count (percentage), as appropriate. Student's *t* test, Wilcoxon rank‐sum test, and chi‐square test were used to compare data between AR patients and controls. Receiver operating characteristic (ROC) curve analysis and the derived area under the curve (AUC) were used to estimate the accuracy of a marker for identifying different subjects. The Spearman rank correlation test was used to perform correlation analysis. SPSS software (version 24.0; IBM Corp., Armonk, NY, USA) and GraphPad Prism 8.01 software (GraphPad Software, La Jolla, CA, USA) were used for statistical analysis and graph making, respectively. Statistical significance was set at *p* < 0.05.

## 3 RESULTS

### 3.1 Characteristics of participants

Among the 120 AR patients, the mean age was 27.9 ± 6.0 years; 54 (45.0%) of these 120 AR patients were men, and 66 (55.0%) were women. Among the 60 controls, the mean age was 28.9 ± 7.6 years; 25 (41.7%) were men, and 35 (58.3%) were women. There was no significant difference in age or sex distribution between patients with AR and controls (*p* > 0.05). Median values of serum IgE, IFN‐γ, IL‐2, IL‐4, and IL‐10 expression were all higher in AR patients than in controls (*p* < 0.001). Among AR patients, the median values of the rhinorrhea score, itching score, sneezing score, congestion score, and TNSS were 2.0 (1.0–3.0), 2.0 (1.3–2.0), 2.0 (2.0–3.0), 2.0 (2.0–3.0), and 8.0 (7.0–9.0), respectively (Table 1).

**TABLE 1:** Participants’ characteristics

Characteristics	Controls (*N* = 60)	AR patients (*N* = 120)	*p* value
Age (years), mean ± SD	28.9 ± 7.6	27.9 ± 6.0	0.721
Gender, No. (%)			0.671
Male	25 (41.7)	54 (45.0)	
Female	35 (58.3)	66 (55.0)	
Serum IgE (IU/ml), median (IQR)	22.3 (15.1–33.2)	304.4 (170.3–512.4)	<0.001
IFN‐γ mRNA, median (IQR)	0.990 (0.640–1.455)	0.455 (0.260–0.640)	<0.001
IL−2 mRNA, median (IQR)	0.995 (0.605–1.373)	0.375 (0.250–0.630)	<0.001
IL−4 mRNA, median (IQR)	0.995 (0.513–1.638)	2.815 (2.013–4.245)	<0.001
IL−10 mRNA, median (IQR)	0.995 (0.690–1.400)	3.000 (1.800–4.085)	<0.001
INSS, median (IQR)			
Rhinorrhea score	‐	2.0 (1.0–3.0)	‐
Itching score	‐	2.0 (1.3–2.0)	‐
Sneezing score	‐	2.0 (2.0–3.0)	‐
Congestion score	‐	2.0 (2.0–3.0)	‐
TNSS, median (IQR)	‐	8.0 (7.0–9.0)	‐

Abbreviations: AR, allergic rhinitis; IFN, interferon; IgE, immunoglobulin E; IL, interleukin; INSS, individual nasal symptom score; IQR, interquartile range; SD, standard deviation; TNSS, total nasal symptom score.

### 3.2 Comparison of lnc‐GAS5, miR‐21, and miR‐140 expression between AR patients and controls

**FIGURE 1:**
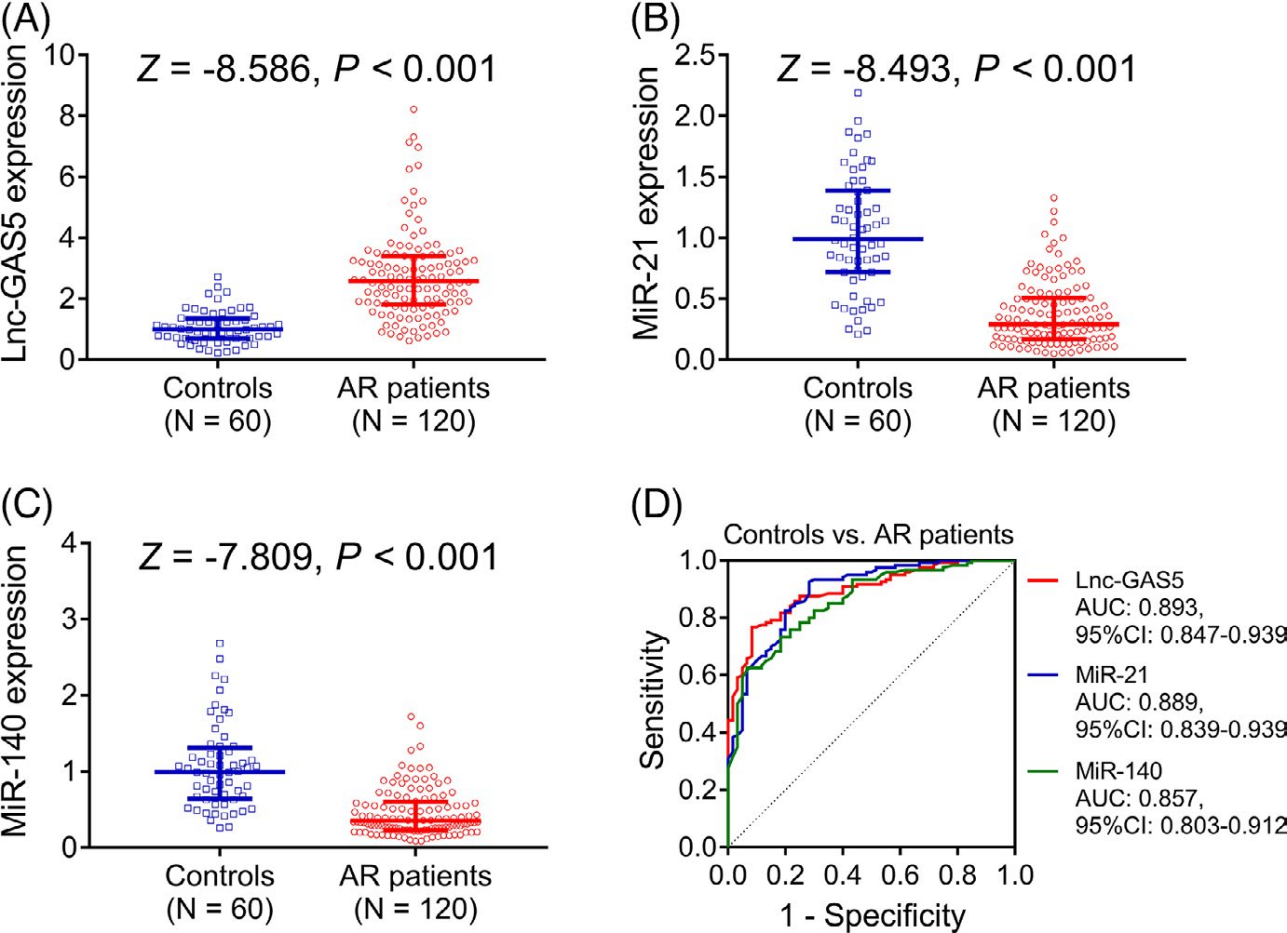
Comparison of the expression of lnc‐GAS5 and its target miRNAs between AR patients and controls. Comparison of lnc‐GAS5 (A), miR‐21 (B), and miR‐140 (C) expression between AR patients and controls; the performance of lnc‐GAS5, miR‐21, and miR‐140 in differentiating AR patients from controls (D). AR, allergic rhinitis; lnc‐GAS5, long non‐coding RNA growth arrest‐specific 5; miR, microRNA; AUC, area under curve; CI, confidence interval

Lnc‐GAS5 expression was higher in AR patients than in controls (median value: 2.580 [1.810–3.408] vs. 0.995 [0.703–1.353]; *p* < 0.001) (Figure 1A). Meanwhile, miR‐21 expression (median value: 0.290 [–0.170–0.508] vs. 0.990 [–0.720–1.388]) and miR‐140 expression (median value: 0.355 [–0.230–0.605] vs. 0.995 [0.640–1.313]) were lower in AR patients than in controls (*p* < 0.001) (Figure 1B and C). Moreover, ROC curves showed that lnc‐GAS5, miR‐21, and miR‐140 all had good potential in discriminating AR patients from controls with AUCs of 0.893 (95% confidence interval [CI]: 0.847–0.939), 0.889 (95% CI: 0.839–0.939), and 0.857 (95% CI: 0.803–0.912), respectively (Figure 1D).

### 3.3 Correlation of lnc‐GAS5 expression with miR‐21 and miR‐140 expression in AR patients

**FIGURE 2:**
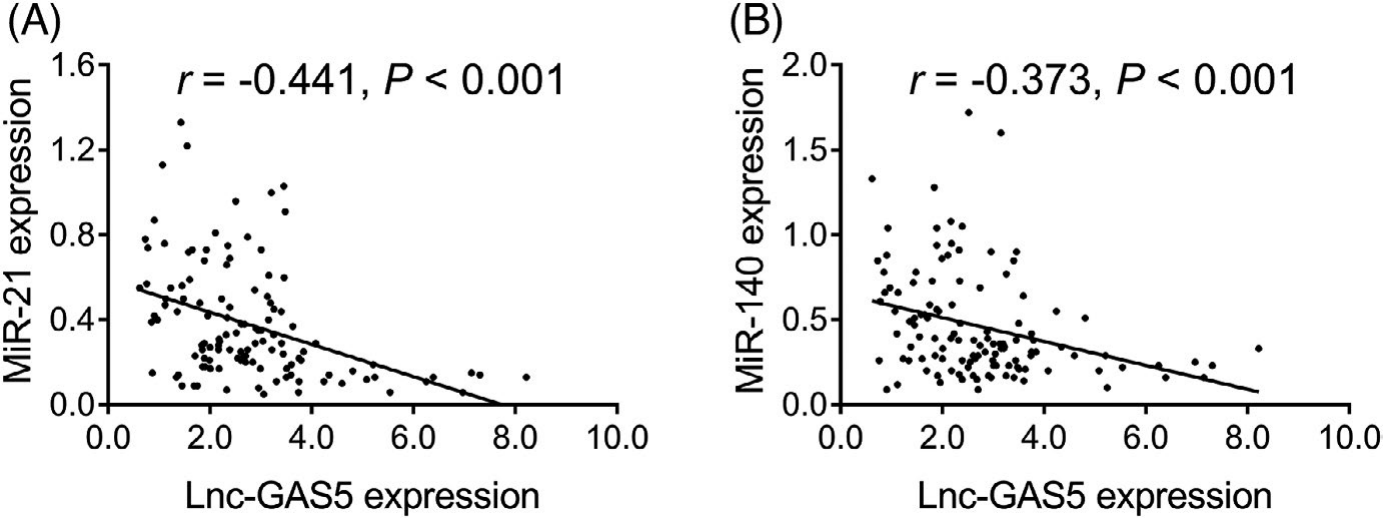
Correlation of expression of lnc‐GAS5 with that of its target miRNAs in AR patients. Correlation between lnc‐GAS5 and miR‐21 expression (A); correlation between lnc‐GAS5 and miR‐140 expression (B). AR, allergic rhinitis; lnc‐GAS5, long non‐coding RNA growth arrest‐specific 5; miR, microRNA

Lnc‐GAS5 expression was negatively correlated with miR‐21 expression (*r* = –0.441, *p* < 0.001) (Figure 2A) and with miR‐140 expression (*r* = –0.373, *p* < 0.001) (Figure 2B).

### 3.4 Correlation of lnc‐GAS5 expression with rhinorrhea, itching, sneezing, and congestion scores and TNSS in AR patients

**FIGURE 3:**
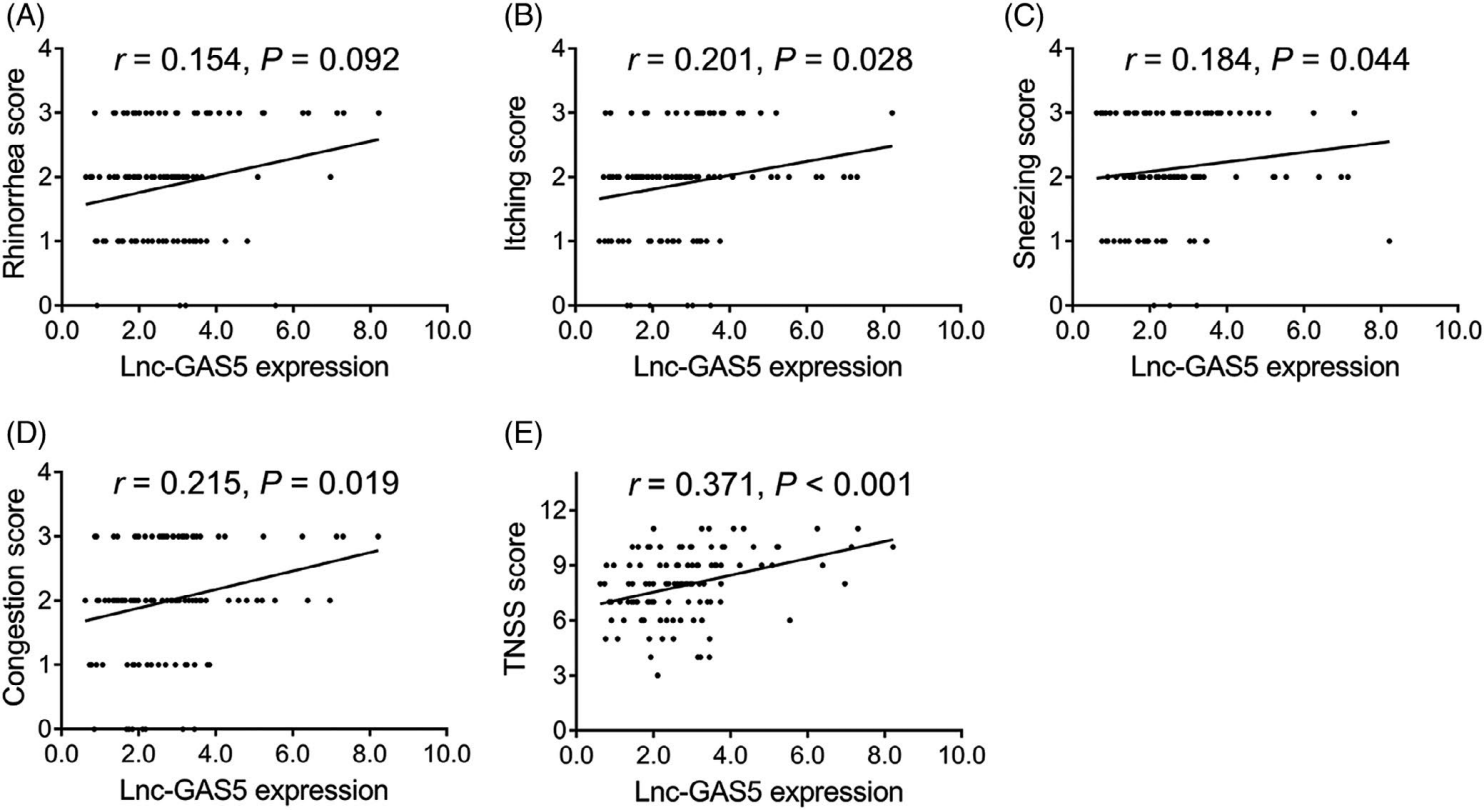
Correlation of lnc‐GAS5 expression with disease severity in AR patients. Correlation of lnc‐GAS5 expression with the rhinorrhea score (A), itching score (B), sneezing score (C), congestion score (D), and Total nasal symptoms scores (TNSS) score (E). AR, allergic rhinitis; lnc‐GAS5, long non‐coding RNA growth arrest‐specific 5; TNSS, total nasal symptom score

Positive associations were found between lnc‐GAS5 expression and the itching score (*r* = 0.201, *p* = 0.028), sneezing score (*r* = 0.184, *p* = 0.044), congestion score (*r* = 0.215, *p* = 0.019), and TNSS score (*r* = 0.371, *p* < 0.001) (Figure 3B‐E); however, no correlation was found between lnc‐GAS5 expression and the rhinorrhea score (*r* = 0.154, *p* = 0.092) (Figure 3A).

### 3.5 Correlation of lnc‐GAS5 expression with IFN‐γ, IL‐2, IL‐4, and IL‐10 expression in AR patients

**FIGURE 4:**
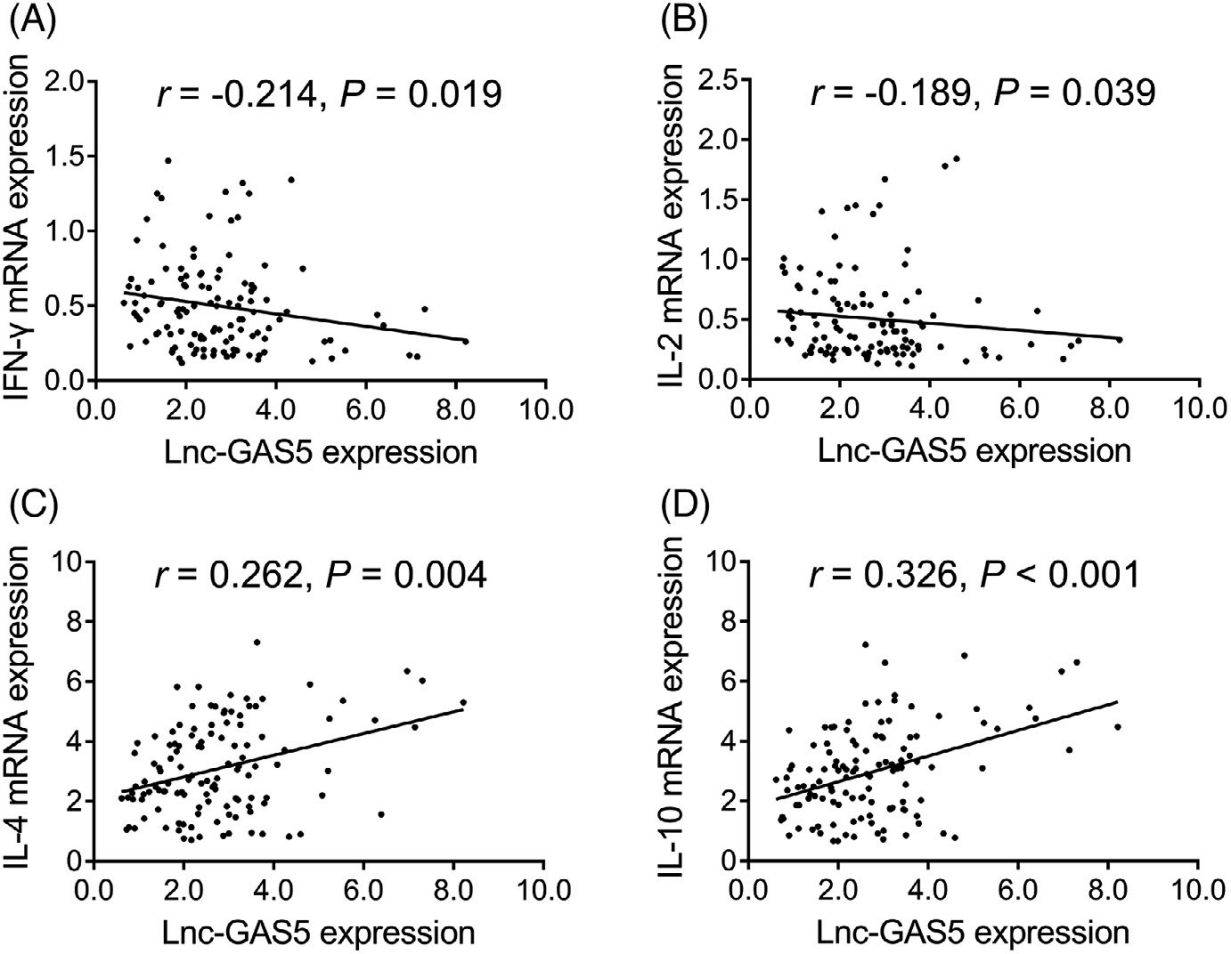
Correlation of lnc‐GAS5 expression with Th1 and Th2 cytokine expression in AR patients. Correlation of lnc‐GAS5 expression with IFN‐γ (A), IL‐2 (B), IL‐4 (C), and IL‐10 (D) expression. AR, allergic rhinitis; lnc‐GAS5, long non‐coding RNA growth arrest‐specific 5; IL = interleukin; IFN, interferon; Th, helper T cell

Lnc‐GAS5 expression was negatively correlated with IFN‐γ (*r* = –0.214, *p* = 0.019) and IL‐2 (*r* = –0.189, *p* = 0.039) expression (Figure 4A and B). However, lnc‐GAS5 expression was positively correlated with IL‐4 (*r* = 0.262, *p* = 0.04) and IL‐10 (*r* = 0.326, *p* < 0.001) expression (Figure 4C and D).

### 3.6 Correlation of miR‐21 expression with disease features and characteristics in AR patients

**FIGURE 5:**
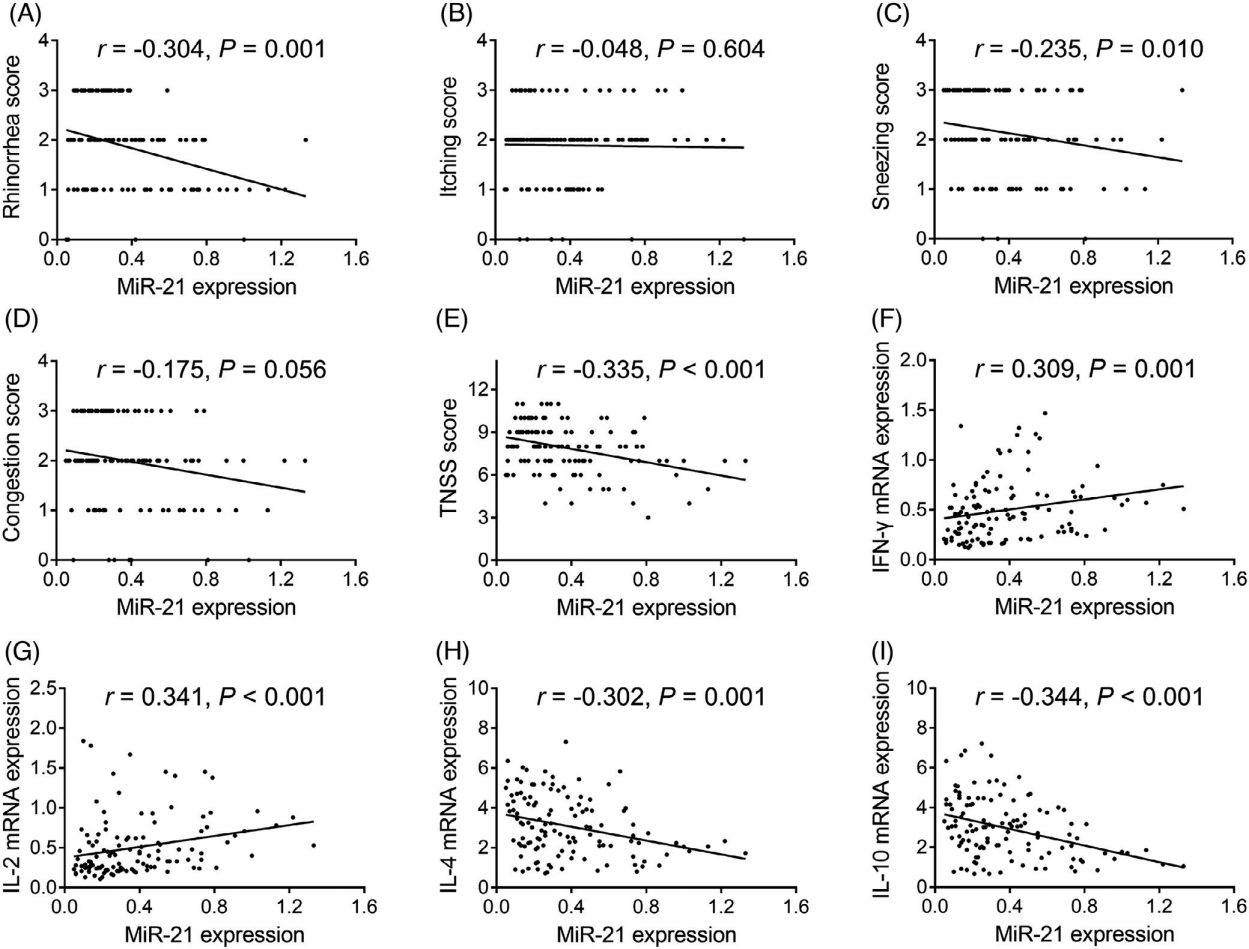
Correlation of miR‐21 expression with disease severity and Th1 and Th2 cytokine expression in AR patients. Correlation of miR‐21 expression with the rhinorrhea score (A), itching score (B), sneezing score (C), congestion score (D), and TNSS (E) as well as IFN‐γ (F), IL‐2 (G), IL‐4 (H), and IL‐10 (I) expression. AR, allergic rhinitis; miR, microRNA; IL, interleukin; IFN, interferon; TNSS, total nasal symptom score; Th, helper T cell

Negative associations were found between miR‐21 expression and the rhinorrhea score (*r* = –0.304, *p* = 0.001), sneezing score (*r* = –0.235, *p* = 0.010), TNSS (*r* = –0.335, *p* < 0.001), IL‐4 expression (*r* = –0.302, *p* = 0.001), and IL‐10 expression (*r* = –0.344, *p* < 0.001) (Figure 5A, C, E, H, and I). Furthermore, miR‐21 expression was positively associated with IFN‐γ expression (*r* = 0.309, *p* = 0.001) and IL‐2 expression (*r* = 0.341, *p* < 0.001) (Figure 5F and G). No correlation was found between miR‐21 expression and itching or congestion scores (*p* > 0.05) (Figure 5B and D).

### 3.7 Correlation of miR‐140 expression with disease features and characteristics in AR patients

**FIGURE 6:**
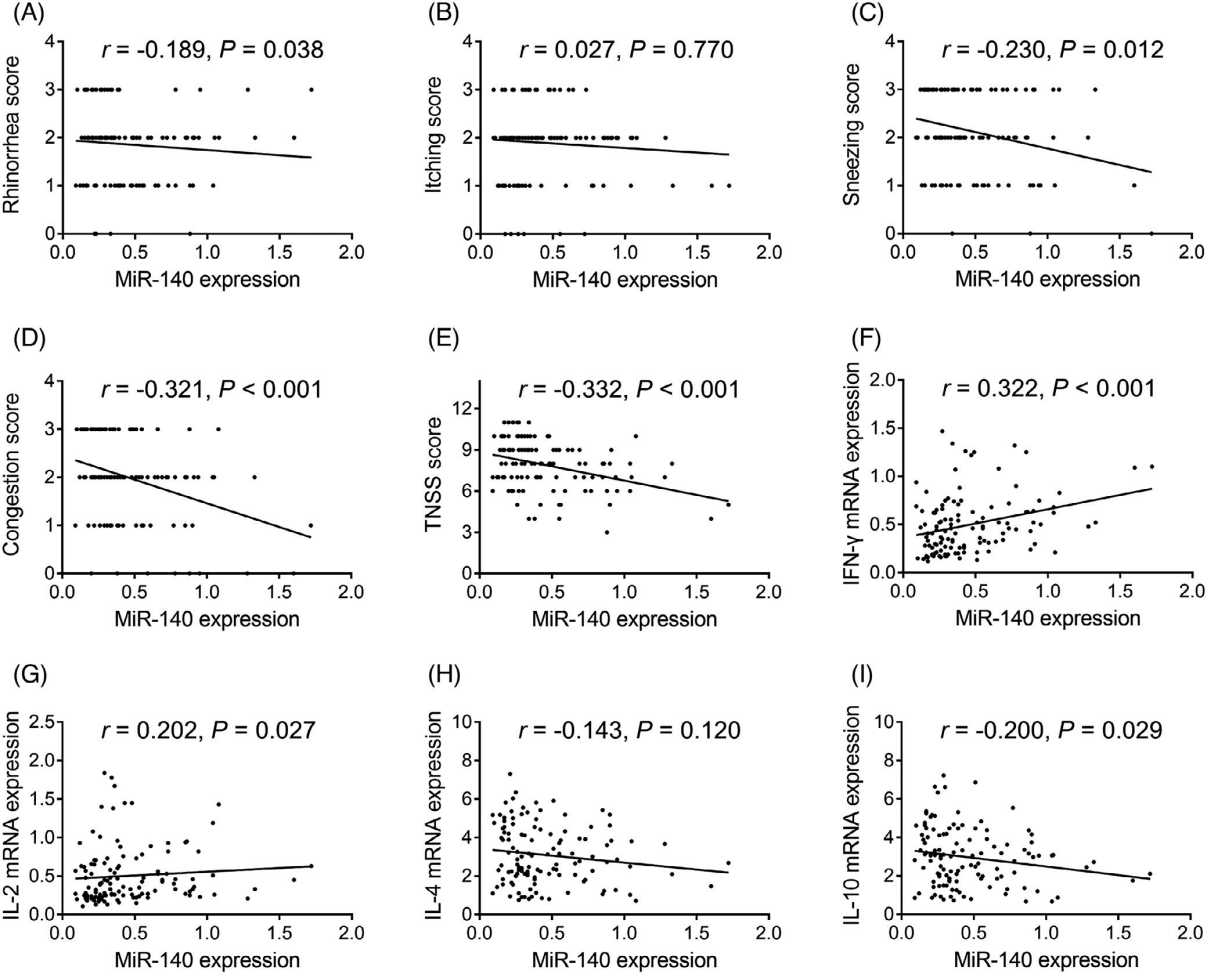
Correlation of miR‐140 expression with disease severity and Th1 and Th2 cytokine expression in AR patients. Correlation of miR‐140 expression with the rhinorrhea score (A), itching score (B), sneezing score (C), congestion score (D), and TNSS (E), as well as IFN‐γ (F), IL‐2 (G), IL‐4 (H), and IL‐10 (I) expression. AR, allergic rhinitis; miR, microRNA; IL, interleukin; IFN, interferon; TNSS, total nasal symptom score; Th, helper T cell

miR‐140 expression was negatively correlated with the rhinorrhea score (*r* = –0.189, *p* = 0.038), sneezing score (*r* = –0.230, *p* = 0.012), congestion score (*r* = –0.321, *p* < 0.001), TNSS (*r* = –0.332, *p* < 0.001), and IL‐10 expression (*r* = –0.200, *p* = 0.029) (Figure 6A, C, D, E, and I). In addition, miR‐140 expression was positively correlated with IFN‐γ expression (*r* = 0.322, *p* < 0.001) and IL‐2 expression (*r* = 0.202, *p* = 0.027) (Figure 6F and G). No correlation was found between miR‐140 expression and itching score or IL‐4 expression (*p* > 0.05) (Figure 6B and H).

## 4 DISCUSSION

With regard to lnc‐GAS5, miR‐21, and miR‐140 expression in immune diseases, previous studies have shown that lnc‐GAS5 expression is increased in T cells in patients with rheumatoid arthritis (RA) compared to that in individuals without RA.^9^ Meanwhile, miR‐140 expression is downregulated in synovial fibrous tissues in RA.^15^ Furthermore, upregulated miR‐21 expression has been detected in patients with ankylosing spondylitis (AS) compared to healthy controls.^16^ In the present study, lnc‐GAS5 expression was found to be elevated, while the expression of its targets, miR‐21 and miR‐140, was decreased in AR patients compared to controls, which is partly in line with previous studies.^9^, ^15^, ^16^ Furthermore, lnc‐GAS5 expression was negatively associated with miR‐21 and miR‐140 expression. There are some potential explanations for these findings. First, the upregulation of lnc‐GAS5 expression could result in an imbalance in Th1 and Th2, which might be involved in the pathogenesis of AR; this would explain the increased expression of lnc‐GAS5 in AR patients.^7^ Second, a decline in miR‐21 expression might be associated with pathogenesis‐related factors of AR, such as transforming growth factor (TGF)‐β and IgE^12^, ^13^; moreover, miR‐140 can modulate Th1 differentiation by targeting signal transducer and activator of transcription 1 (STAT1), which might also be correlated with the pathogenesis of AR.^7^, ^17^ This would explain the reduced expression of miR‐21 and miR‐140 in AR patients. Third, lnc‐GAS5 can target miR‐21 and miR‐140 in patients with immunological disorders, such as HIV and recurrent pregnancy loss^10^, ^11^; a similar targeting mechanism may also exist in AR, which would explain the negative association between lnc‐GAS5 expression and miR‐21 and miR‐140 expression.

Regarding the correlation between lnc‐GAS5 expression and immune‐disease severity, a positive correlation has been found between lnc‐GAS5 expression and the Expanded Disability Status Scale score for multiple sclerosis.^18^ However, the correlation between lnc‐GAS5 expression and AR severity of AR remains unclear. In our study, we found that lnc‐GAS5 expression was positively correlated with AR severity. This result could be explained by lnc‐GAS5‐related inhibition of Th1 differentiation and increase in Th2 differentiation *via* Enhancer of Zeste Homolog 2 (EZH2) and T‐bet, which would decrease immune function, promote inflammation, and consequently aggravate AR severity.^7^, ^19^ The aforementioned data indicate that lnc‐GAS5 may serve as a biomarker of AR severity.

Previous studies have reported that lnc‐GAS5 is associated with an imbalance in Th1 and Th2 in immune disorder‐related diseases, such as recurrent pregnancy loss.^11^ Thus, we speculated that lnc‐GAS5 expression might be correlated with Th1 and Th2 cytokine expression in AR. We observed that lnc‐GAS5 expression was negatively associated with IFN‐γ and IL‐2 expression and positively correlated with IL‐4 and IL‐10 expression. It is possible that lnc‐GAS5 could downregulate T‐bet and EZH2 to suppress Th1 differentiation and promote Th2 differentiation, consequently decreasing IFN‐γ and IL‐2 expression and increasing IL‐4 and IL‐10 expression.^7^

Regarding the association of miR‐21 and miR‐140 with the severity of immune diseases, a previous study has demonstrated that miR‐21 expression is negatively associated with chronic health evaluation II and sequential organ failure assessment scores in sepsis^20^; a negative correlation was also found between miR‐140 expression and disease severity in multiple sclerosis.^17^ In our study, we found that among AR patients, expression of both miR‐21 and miR‐140 was negatively correlated with AR severity and Th2 cytokine expression and positively correlated with Th1 cytokine expression. It is possible that miR‐21 decreases the immune response by affecting T cell polarization, which could lead to more severe AR^19^, ^21^; besides, miR‐140 can modulate the STAT1 and T‐bet signaling pathways, which may regulate Th1 differentiation and aggravate AR severity.^17^, ^19^ This would explain the negative correlation between the expression of miR‐21 and miR‐140 and the severity of AR. Furthermore, it is possible that miR‐21 and miR‐140 upregulate Th1 differentiation but downregulate Th2 differentiation by targeting GATA‐3 and T‐bet.^17^, ^22^, ^23^ This would explain the positive correlation of miR‐21 and miR‐140 expression with Th1 cytokine expression as well as the negative correlation of miR‐21 and miR‐140 expression with Th2 cytokine expression.

There were several limitations in our study. First, the sample size was relatively small, which could result in insufficient statistical power. Second, the study did not explore the association of lnc‐GAS5 and its targets (miR‐21 and miR‐140) with the prognosis of AR, such as treatment response and recurrence; therefore, longer‐term observation is needed in future studies. Third, we only detected the expression of lnc‐GAS5 and its targets (miR‐21 and miR‐140) as well as the expression of Th1/Th2 cytokines in nasal‐mucosa tissue samples; analysis of their expression in other sources of samples, such as the blood, to verify their correlation with AR is required. Fourth, the clinical value of lnc‐GAS5, miR‐21, and miR‐140 expression in blood samples of AR patients should be explored in future studies. Finally, the mechanisms underlying the roles of lnc‐GAS5, miR‐21, and miR‐140 in the pathogenesis and progression of AR should be investigated in the future.

In conclusion, the expression of lnc‐GAS5, miR‐21, and miR‐140 was correlated with disease risk, symptom severity, and Th1/Th2 cytokine expression in AR, indicating that lnc‐GAS5, miR‐21, and miR‐140 may be potential biomarkers for AR.

## CONFLICT OF INTEREST

Authors declared that they have no competing interests.

## Supporting information

Table S1

## Data Availability

Data sharing is not applicable to this article as no datasets were generated or analyzed during the current study.
