# Supplementary material for: Long non‐coding RNA growth arrest‐specific 5 and its targets, microRNA‐21 and microRNA‐140, are potential biomarkers of allergic rhinitis
Source: J Clin Lab Anal. 2021 Sep 2;35(10):e23938. doi: 10.1002/jcla.23938 (PMC8529140; doi:10.1002/jcla.23938)
Supplement: Supplementary file 1 — Table S1 [file JCLA-35-e23938-s001.docx]

**Supplementary table 1.** Primers

| Gene | Forward (5'->3') | Reverse (5'->3') |
| --- | --- | --- |
| Lnc-GAS5 | GGACCGGGAGATAGGAGTG | CACGGACTCCAGGTGATGAG |
| MiR-21 | ACACTCCAGCTGGGTAGCTTATCAGACTGA | TGTCGTGGAGTCGGCAATTC |
| MiR-140 | AAAGGACTGCAGCAACAACC | CCATTGAGCATCACATGGAC |
| IFN-γ | GGTCATTCAGATGTAGCGG | CACTCTCCTCTTTCCAATTC |
| IL-2 | AAACTCACCAGGATGCTCAC | TGTTTCAGATCCCTTTAGTTCCAG |
| IL-4 | AGCAGTTCCACAGGCACAAG | CTCTGGTTGGCTTCCTTCACA |
| IL-10 | TGTTGCCTGGTCCTCCTGACT | GCCTTGATGTCTGGGTCTTGGTT |
| GAPDH | TGACCACAGTCCATGCCATCAC | GCCTGCTTCACCACCTTCTTGA |
| U6 | CTCGCTTCGGCAGCACATATACTA | ACGAATTTGCGTGTCATCCTTGC |

Lnc-GAS5, long non-coding growth arrest specific 5; MiR, microRNA; IFN, interferon; IL, interleukin.
